# Macrophage Immunomodulation: The Gatekeeper for Mesenchymal Stem Cell Derived-Exosomes in Pulmonary Arterial Hypertension?

**DOI:** 10.3390/ijms19092534

**Published:** 2018-08-27

**Authors:** Gareth R. Willis, Angeles Fernandez-Gonzalez, Monica Reis, S. Alex Mitsialis, Stella Kourembanas

**Affiliations:** Division of Newborn Medicine, Boston Children’s Hospital and Department of Pediatrics, Harvard Medical School, Boston, MA 02115, USA; gareth.willis@childrens.harvard.edu (G.R.W.); Angeles.Fernandez-Gonzalez@childrens.harvard.edu (A.F.-G.); monica.reis@childrens.harvard.edu (M.R.); Alex.Mitsialis@childrens.harvard.edu (S.A.M.)

**Keywords:** exosomes, extracellular vesicles (EVs), pulmonary hypertension (PH), bronchopulmonary dysplasia (BPD), mesenchymal stem cells (MSCs), MSC exosomes (MEx), pulmonary arterial hypertension (PAH), macrophages, inflammation

## Abstract

Pulmonary arterial hypertension (PAH) is a progressive disease characterized by remodeling of the pulmonary arteries, increased pulmonary infiltrates, loss of vascular cross-sectional area, and elevated pulmonary vascular resistance. Despite recent advances in the management of PAH, there is a pressing need for the development of new tools to effectively treat and reduce the risk of further complications. Dysregulated immunity underlies the development of PAH, and macrophages orchestrate both the initiation and resolution of pulmonary inflammation, thus, manipulation of lung macrophage function represents an attractive target for emerging immunomodulatory therapies, including cell-based approaches. Indeed, mesenchymal stem cell (MSC)-based therapies have shown promise, effectively modulating the macrophage fulcrum to favor an anti-inflammatory, pro-resolving phenotype, which is associated with both histological and functional benefits in preclinical models of pulmonary hypertension (PH). The complex interplay between immune system homeostasis and MSCs remains incompletely understood. Here, we highlight the importance of macrophage function in models of PH and summarize the development of MSC-based therapies, focusing on the significance of MSC exosomes (MEx) and the immunomodulatory and homeostatic mechanisms by which such therapies may afford their beneficial effects.

## 1. Introduction

Pulmonary arterial hypertension (PAH) is a rare, chronic disease characterized by remodeling of the pulmonary arteries, increased pulmonary infiltrates, elevated pulmonary vascular resistance, and right ventricular hypertrophy (RVH) [1,2,3]. Such characteristics can manifest into the clinical presentation of syncope, shortness of breath upon exercise, chest pain, and fatigue [4]. Defined as a sustained elevation of pulmonary arterial pressure >25 mmHg at rest or >30 mmHg with exercise, with a mean pulmonary-capillary wedge pressure and left ventricular end-diastolic pressure of <15 mmHg, PAH includes a heterogeneous group of clinical entities that share similar histological and pathological changes [5]. Progressive elevations in mean pulmonary arterial pressure (mPAP) eventually gives rise to right heart failure, where the prognosis is ominous. In turn, PAH remains a fatal disease with, at present, a five-year survival rate of less than 60% at the time of diagnosis [6].

Despite a complex multi-pathogenesis, over the past two decades an improved molecular understanding in PAH pathophysiology has led to the development of new therapeutic options. In patients with PAH, the identification of dysfunctional endothelial cells and an imbalance in the molecular modulators of the pulmonary vasculature have become the chief targets for pharmacological agents [5,7,8]. In turn, the introduction of pulmonary vasodilators that target nitric oxide (NO), endothelin-1, and/or prostaglandin signaling pathways have slowed disease progression and afforded a better quality of life for patients [9,10], although, such medicinal products often have a variable impact depending on the etiology of PAH [11]. Arguably, the complex molecular interplay between immune cell subsets and vascular cells in the pathophysiology of PAH remains poorly understood and has contributed to a paucity in successful therapies.

Altered immunity and inflammation are increasingly recognized as core features of PAH, where synergistic interplay between various infiltrating and inflammatory cells such as T and B lymphocytes, dendritic cells (DCs), mast cells, monocytes, and macrophages play a central role in the pathogenesis [3,11,12,13]. As our current understanding of PAH immunology improves, such advances may pave the way for the ‘next generation’ of therapeutics. To this end, immunomodulatory approaches have been considered, where cell based-therapies have shown increasing promise in preclinical models of pulmonary hypertension (PH). In this review, we summarize our current understanding of macrophage-mediated inflammation in PAH. We will highlight the developments of novel cell-based approaches in preclinical models of PH, focusing on mesenchymal stromal/stem cell (MSC)-based therapeutics, in particular MSC exosomes/extracellular vesicles, and the immunomodulatory mechanisms by which such therapies may afford their beneficial effects.

## 2. Macrophage-Mediated Immunity in PAH

Growing histological and biomarker evidence demonstrates that early and persistent inflammation is a critical component of PAH, with several studies observing circulating autoantibodies, elevated levels of macrophage inflammatory protein-1α, and increased pro-inflammatory cytokines levels such as interleukin-1 (IL-1), IL-6, and tumor necrosis factor (TNF)-α in experimental and clinical PAH [3,14]. By convention, herein animal models are referred as having PH rather than PAH. Among the inflammatory cells implicated in PAH, those of the monocyte/macrophage lineage have been often correlated with disease severity and progression. For example, in experimental PH and clinical PAH, CD68^+^ macrophages are predominant in advanced obliterative plexiform lesions [3,15,16,17]. Their recruitment to perivascular areas is instigated by dysfunctional pulmonary endothelial cells, through increased macrophage migration inhibitory factor (MIF) [18,19], or reduced bone morphogenetic protein receptor type 2 (BMPR2) signaling [20]. Pulmonary macrophage influx and activation is also mediated by leukotriene B_4_ (LTB_4_), a lipid mediator produced by multiple cells in the lung. Macrophage-derived LTB_4_ promotes endothelial injury and pulmonary artery (PA) smooth muscle cell (SMC) proliferation in PAH patients and rat models of PH [21,22]. Interestingly, blocking macrophage-derived LTB_4_ biosynthesis or signal transduction reverses experimental PH, and depleting CD68^+^ macrophages prevents experimental PH [21]. Using a murine model of hypoxia-induced PH we have demonstrated that an early and transient phase of hypoxic inflammation, characterized by ‘alternative’ macrophage activation and the upregulation of inflammatory markers, is essential for vascular remodeling and the subsequent establishment of PH [23]. Here, in the bronchoalveolar lavage fluid (BALF) of hypoxic animals, resident pulmonary macrophages (CD11c, F4/80^+ve^) occupied an ‘alternatively’ activated macrophage phenotype defined by elevated levels of arginase-1 (Arg1), found in inflammatory zone-1 (FIZZ) and chitinase-like 3 (Chi3l3). Compared to control animals, the upregulation of IL-1β, macrophage inflammatory protein 1-α (MIP-1α), IL-17, IL-2, as well as IL-13 and IL-4 has been observed in as early as two days of hypoxia exposure (8.5% O_2_). Moreover, studies have shown that the suppression of this early inflammatory response can prevent the development of RVH in models of hypoxia-induced PH [23,24]. Concurrently, it has been demonstrated that macrophage activation can also be driven by adventitial fibroblasts through paracrine signaling involving Il-6, signal transducer, and activator of transcription 3 (Stat3), hypoxia-inducible factor 1 (Hif1) and CCAAT enhancer binding protein beta (C/EBPB) [25].

Typically, alveolar and interstitial resident macrophages maintain an anti-inflammatory microenvironment that, at baseline, is not permissive to the recruitment and self-renewal of circulating monocytes, but in response to injury, recruited monocytes can efficiently respond to local environmental signals, differentiate, and polarize to different activation states. The “reprogramming” of monocytes over the course of injury (initiation/progression) results in a cell with a different epigenomic and transcriptomic landscape that can differentially localize to specific areas of the lung. Recent, elegant studies in preclinical models stress the effects of the microenvironment on monocytic differentiation and address the diversity of temporal and compartmental macrophage phenotypes in relation to lung homeostasis and disease [26]. Arguably, the classic ‘M1/M2’ binary approach is an over simplification that is perhaps considered obsolete [27]. In our hands, using models of hypoxia-induced PH and in experimental bronchopulmonary dysplasia (BPD), both BALF and whole lung samples display upregulated markers of both pro-inflammatory “M1” and alternative activation “M2”, which in agreement with the concept of a continuum in macrophage activation as opposed to a dichotomous paradigm [28,29,30]. Notwithstanding such limitations, we have previously shown that dysregulated “M2-like” macrophages from chronic exposure to hypoxia are also able to adopt a ‘pro-remodeling’, alternatively-activated phenotype that induces pulmonary artery smooth muscle cell (PASMC) proliferation [23]. In accordance with that finding, others have reported the temporal and spatial distribution of macrophage activation by adventitial fibroblasts in hypoxia-induced PH and patients with PAH. In these conditions macrophages participate in vascular remodeling and fibrotic tissue responses [25,26,31,32].

Numerous efforts have been made to further characterize the role of macrophages and other myeloid cells in the initiation and maintenance of pulmonary vascular remodeling. In line with this, an essential role for these cells in the pathogenesis of PH is perhaps best demonstrated in experiments in which in vivo depletion of monocytes/macrophages attenuated pulmonary vascular remodeling. Previous studies have found that depletion of circulating monocytes by administration of liposomal clodronate, prevented pulmonary vessel remodeling in a rat model of hypoxia-induced PH [33]. Moreover, the depletion of recruited monocytes with gadolinium chloride in neonatal hyperoxia-exposed rats demonstrated reduced RVH and decreased SMC proliferation [34]. Recently, Zaloudikova et al. demonstrated that depletion of alveolar macrophages attenuates hypoxic PH, emphasizing the role of pulmonary macrophages in PH pathogenesis [35]. Like pulmonary resident macrophages, myeloid-derived suppressor cells (MDSCs) are a different type of myeloid cell with immuno-modulatory activity. Interestingly, recent studies performed in murine models of PH and pulmonary fibrosis have demonstrated that macrophage depletion (by using clodronate liposomes) augmented pulmonary pressure secondary to elevated circulating levels of MDSCs. In turn, the MDSC expansion in the bone marrow and infiltration into the lungs coordinate the inflammatory milieu that leads to pulmonary vascular remodeling [36].

Pulmonary monocyte infiltration, macrophage polarization, and vascular remodeling are controlled by chemokines such as C-C motif chemokine ligand 5 (CCL5), CCL2, and C-X3-C motif chemokine ligand 1 (CXC3CL1) and their cognate receptors. The pharmacological or genetic ablation of such chemokines and their receptors have been relevant to the pathogenesis of PH [3,37]. Recently, Florentin and colleagues investigated the role and mobilization of blood-borne circulating monocytes and their contribution to arteriolar wall remodeling [38]. They showed that genetic or pharmacological deficiency of C-X3-C motif chemokine receptor 1 (Cx3cr1)-expressing monocytes limited the recruitment of monocytes and the development of lung remodeling in hypoxia- and monocrotaline (MCT)-induced PH without amelioration of vascular hemodynamics [38]. Conversely, other studies have found a prominent role for Cx3cr1 in modulating macrophage polarization, PA-SMC proliferation, and altered hemodynamics without concomitant decreases in lung monocyte counts [39]. Genetic disruption and pharmacological inactivation of C-C chemokine receptor 5 (CCR5), a chemokine expressed on macrophages and vascular cells, decreased PA-SMC proliferation and recruitment of perivascular and alveolar macrophages during hypoxia exposure in mice [40]. Moreover, conflicting studies have shown that CCL2-CCR2 signaling has no significant effect [39] or a positive effect on monocyte recruitment and pulmonary artery wall remodeling [38]. Collectively, it appears that the macrophage is at the epicenter of immune responses in the lung. However, it is important to note that although histological and physiological benefits have been witnessed in most, albeit not all, of the studies that deplete endogenous monocyte/macrophage populations, indices such as inflammation, vascular remodeling and right ventricle (RV) abnormalities seldom return to ‘baseline’. Although macrophage depletion techniques such as clodronate administration/dosing render their own limitations where they often only eliminate partial monocyte/macrophage populations [32], it is fair to postulate that targeting macrophages in isolation may only yield limited therapeutic benefits. Furthermore, in addition to macrophages it is well known that various immunologically active cells such as T and B lymphocytes, DCs, and innate lymphoid cells (ILCs) play a central role in the pathogenesis of PAH (reviewed in [3,11,12,13,41,42,43]). On balance, although this review focuses on the role of macrophages in the context of PAH, the influence of other immune cells and their subsequent interplay with macrophages is intrinsic to the pathophysiology of PAH. 

## 3. Interplay of Monocytes/Macrophages with Other Immune Cell Types in PAH

Macrophage mediated-inflammation is complex and crosstalk between macrophages can shape the regulatory properties of the lung milieu in a direct and indirect fashion [27,30,44,45,46]. In the lung, the innate and adaptive immune system work synergistically to maintain tissue homeostasis. Both resident and monocyte-derived macrophages (in particular interstitial macrophages) readily interact with resident T lymphocytes to orchestrate an adaptive immune response by directly modulating the activation of lung antigen presenting cells [47,48]. To maintain pulmonary homeostasis, interstitial macrophages constitutively produce anti-inflammatory cytokine IL-10 through the activation of the Toll-like receptor 4 (TLR_4_)/Myeloid differentiation primary response 88 (MyD88) signaling pathway [49]. It is well accepted that the secretion of IL-10 by interstitial macrophages inhibits DC maturation and migration, thus preventing the development of DC-driven T helper type 2 (Th2) and Th17 inflammatory responses in the lung [49,50]. Depletion of CD4^+^ T cells, antigen-specific Th2 response, or the pathogenic Th2 cytokine Il-13 have been shown to ameliorate pulmonary arterial muscularization. In addition, macrophage-derived IL-10 drives the polarization of CD4^+^ T cells into a regulatory phenotype characterized by the expression of the transcription factor FoxP3 [51,52]. In turn, these regulatory T cells play a pivotal role in maintaining pulmonary homeostasis, where they prevent vascular endothelial injury and serve to protect against adverse ventricular remodeling, contributing to improved cardiac function [53].

Macrophages represent important local sources of factors that regulate pulmonary vascular remodeling. Although it is difficult to establish a temporal order in the inflammatory events leading to the pathogenic functions of these different subsets of myeloid cells, their relative contributions to the initiation and maintenance of pulmonary vascular remodeling is critical in the development of cell-based immune therapies. More research investigating the immune/inflammatory component of PAH is clearly needed. However, driving macrophages towards an anti-inflammatory and regenerative phenotype is considered a potential therapeutic strategy to halt and even reverse disease progression in cardio-respiratory disorders where inflammation prevails.

## 4. Mesenchymal Stem/Stromal Cell (MSC) Based-Therapies in Experimental PH

Stem and progenitor cell based-therapies have shown promise in treating several experimental models of lung disease relevant to PAH. Transplantation of different stem/progenitor cell types, for example, MSCs [54,55,56,57], induced pluripotent stem cells (iPS) [58], endothelial progenitor cells (EPCs) [59,60,61], and human amnion epithelial cells (AECs) [62], have effectively prevented, treated, and/or reversed core features of experimental PH and associated lung diseases where RVH occurs. Such cell-based therapies have been effective in providing protective, anti-inflammatory, regenerative, and improved functional outcomes (reviewed in [63,64,65]). Herein, we will focus on the application of MSC-based approaches in experimental PH.

MSCs are non-hematopoietic adult ‘stem’ cells. Methods for the in vitro propagation of MSCs from several human tissues, including bone marrow, Wharton’s jelly, umbilical cord blood, and adipose tissue are well established [66,67]. MSCs are defined by their adherence to plastic, a baseline differentiation potential to osteocytes, chondrocytes, and adipocytes in vitro, and by the presence of widely accepted surface markers (as defined by the International Society for Cellular Therapy [68]). There is a burgeoning awareness in the potential therapeutic applications of MSCs, since their immunomodulatory capabilities have been shown to be effective in ameliorating injury and/or reestablishing homeostasis in several preclinical models of lung disease such as acute lung injury [69], pulmonary fibrosis [70,71], chronic obstructive pulmonary disorder (COPD) [72,73], and asthma [74]. MSCs are attractive candidates for allogenic therapies. They boast an established/growing safety profile, and the immunomodulatory potential has been previously demonstrated in approved clinical protocols where allogenic MSC therapy is used for the clinical management of steroid refractory acute graft-versus-host disease (aGvHD) [75] and is in clinical development for numerous cardio-respiratory disorders [70,76,77,78].

In models of MCT-induced and overflow-induced PH, intravenous (IV) administration of bone marrow-MSCs or umbilical cord blood-MSCs ameliorates core disease features, reducing indices of RVH (Fulton’s index) and improving right ventricular ejection fraction (RVEF), respectively [79,80]. Similar results have been reported for intratracheal MSC administration [57]. We have previously demonstrated the therapeutic capacity of MSC-expressing heme oxygenase-1 (HO-1), which is known to have a protective function and to restore homeostasis in many diseases [24]. Here, transplantation of MSCs expressing HO-1 in a chronic hypoxia-induced PH model blunted the hypoxia-induced elevation in right ventricular systolic pressure (RVSP) and dramatically reduced RVH. Interestingly, Kanki-Horimoto et al. intravenously administered MSCs overexpressing endothelial nitric oxide synthase (eNOS) in MCT-induced PAH rats [56]. They found that the RVSP and RV/body weight (RV:BW) were lower in the eNOS-MSC experimental group compared to that or the animals that received ‘naïve’ MSCs. Similarly, in a neonatal murine model of hyperoxia-induced BPD, a chronic lung disorder associated with secondary PH, we [81] and others [82,83] have shown that a bolus dose of MSCs can dampen whole lung and BALF inflammation, effectively lower the RV/left ventricle (LV)+septum ratio and reduce RVSP. Collectively, MSC-therapy has been shown to reduce lung inflammation, ameliorate vascular remodeling, and improve hemodynamic function in several models of experimental PH and BPD.

It should be noted that for the purpose of this manuscript we have focused on the results regarding the RV outcomes. However, RV outcomes and vascular muscularization represent one type of injury among several injurious events to the pulmonary artery that may collectively contribute to PAH. Notwithstanding such limitations, whether the improvement of RV physiology was due to a direct effect of the MSC therapy or the decrease in pulmonary resistance remains unclear. On this note, with several studies providing little evidence of significant engraftment of MSCs in recipient animals, arguably the RV has never been directly ‘targeted’ by IV MSC administration [24,84]. In turn, such findings imply that perhaps the immunomodulatory and therapeutic mechanism of action for MSCs are paracrine in nature. Indeed, numerous studies have demonstrated that MSCs have the ability to secrete paracrine factors, which they can mitigate tissue damage [84]. Interestingly, in experimental BPD models, our group [55,81] and others [83] have previously shown that the MSC-derived cell-free conditioned media afforded superior protection compared to MSCs themselves in preventing alveolar loss. In an extension of these studies, using neonatal mice exposed to 75% O_2_ from post-natal day-1 (PND1) to PND14 to initiate lung injury, we demonstrated that administration of the conditioned media from mouse bone marrow-MSCs reversed hyperoxia-induced pulmonary fibrosis and peripheral devascularization, improved lung alveolar development, and fully reversed PH and RVH, compared to control animals that received an equivalent dose of mouse lung fibroblast conditioned media [55]. Importantly, more recent studies have shown that one of the ‘chief’ therapeutic vectors within MSC conditioned media is represented by exosomes [28,29].

## 5. Application of MSC-Exosome (MEx) Therapy in Experimental PH

**Figure 1:**
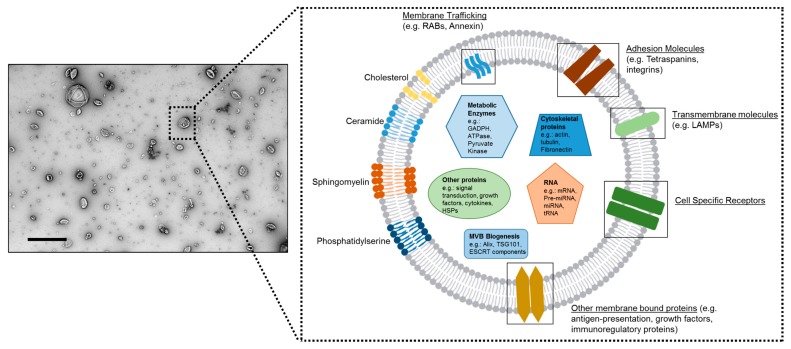
Mesenchymal stem/stromal cell-exosomes (MEx). (**Left panel**) Transmission electron microscopy (TEM) image of human umbilical Wharton’s jelly-derived MEx. Scale bar = 500 nm. (**Right panel**) Schematic representation of MEx composition. MEx contain molecules associated with the pathways of their biogenesis, such as small G-proteins RABs, tumor susceptibility gene 101 (TGS101), programmed cell death 6 interacting protein (Alix), Syntenin, Annexins, and Flotillin 1 (FLOT1). MEx’s cargo includes small non-coding RNAs, but also macromolecular modules, growth factors, and metabolic enzymes. Although heterogenous by nature, typically, exosomes have a diameter of 35–150 nm. Adapted from Willis et al. [96] and Willis et al. [28]. Multivesicular bodies (MVB); endosomal sorting complexes required for transport (ESCRT); glyceraldehyde 3-phosphate dehydrogenase (GAPDH); lysosome-associated membrane glycoprotein (LAMPs).

Several studies have reported that the therapeutic capacity of MSCs is harnessed in their secretome, and more recent studies have demonstrated that the ‘major’ therapeutic vector therein is represented by the exosomes. Extracellular vesicles (EVs) are submicron, plasma membrane enclosed signaling vectors that represent a highly effective means of cell-to-cell communication [85]. During their biogenesis, EVs and more specifically the EV subtype secreted through the endosomal pathway (exosomes), engulf part of their parental cell, becoming enriched in an array of bioactive cargo. Such cargo has been reported to include genetic information (such as small noncoding RNAs), free fatty acids, surface antigens, and protein (reviewed in [85,86,87,88,89,90,91], Figure 1). Albeit complex and incomplete, an improved understanding in EV biology coupled with advances in EV purification and characterization has been paralleled with a broadening realization of the diverse role that EVs play in health and disease. As such, in addition to a prominent diagnostic and prognostic role, exosomes, and for the context of this review, specifically MSC exosomes (MEx), now represent novel therapeutic reagents across a spectrum of disciplines. Perhaps as a consequence of a ‘relatively’ new multi-disciplinary research field, we are often held ransom to the nomenclature and classification of EVs [92]. Thus, for the purposes of this manuscript, herein we will adopt the nomenclature chosen by the cited articles, and support nomenclature with the respective EV isolation methods where appropriate. It is also important to acknowledge that the MSC mechanisms of action may not be restricted to strict paracrine mechanisms that involve exosomes but may also involve transfer of higher-complexity components, including mitochondria, either by larger microvesicles (>500 nm in diameter) or by cell-to-cell contact [93,94,95]. In the context of experimental BPD and PH, in our hands, the efficacy of purified exosome administration is equal to or even superior to that of live MSCs. However, the possibility that certain pathologies could respond better (or even exclusively) to live MSC therapies cannot be ignored, and any conjectures on the topic can only be speculative at present.

## 6. MSC-Exosome (MEx) Therapy Ameliorates Core Physiological Features of Experimental PH

The therapeutic capacity of exosomes generated by MSCs have been tested in diverse preclinical models [97,98,99,100,101,102] (MEx therapy has recently been reviewed [103]). Notably, MEx have afforded significant functional benefits in hypoxia-, hyperoxia-, MCT- and vascular endothelial growth factor receptor antagonist SUGEN5416 (SU)/hypoxia-induced models of PH (summarized in Table 1). Using a murine model of hypoxia-induced PH, our group has demonstrated that exosomes mediate the cytoprotective effect of bone marrow derived-MSCs [29]. Here, administration of MEx, isolated by size exclusion chromatography, identified through widely accepted EV markers, and visualized by electron microscopy, protected against the elevation of RVSP and the development of RVH after three weeks of hypoxic (8% O_2_) exposure. Importantly, exosome-depleted conditioned media had no effect. Moreover, MEx treatment was also able to abrogate the early hypoxic macrophage pulmonary influx and down-regulate hypoxia-activated inflammatory pathways, thus mediating the anti-inflammatory properties of MSCs. More recently, using a neonatal hyperoxia model of BPD we were the first to show that a bolus IV dose of ‘purified’ MEx derived from either human umbilical cord Wharton’s jelly or human bone marrow, significantly improved lung morphology and pulmonary development, decreased lung fibrosis, and ameliorated pulmonary vascular remodeling RVH and RV:BW ratio [28]. Notably, in this study, MEx were isolated from conditioned media by differential centrifugation coupled with tangential flow filtration before further purification using an Opti-prep™ cushion gradient. The exosome containing fraction of the cushion gradient occupies a typical density of ~1.18 g/mL and boasts a low protein:vesicle ratio, indicating high purity.

In a carefully designed study, Aliotta and colleagues found that administration of MEx prevented and reversed MCT-induced PH, characterized by ameliorating elevations in RV/LV+septum ratio and pulmonary vasculature remodeling [104]. Furthermore, they noted MEx contain increased levels of microRNAs that may blunt angiogenesis, inhibit proliferation of neoplastic cells, and induce senescence of vascular SMCs and endothelial progenitor cells (EPCs). This suggests that the genetic cargo MEx may be, at least in part, responsible for such beneficial effects. However, on interpretation of such data, it is important to consider the purity of the EV preparations. As an emerging field, EV isolation, purification, and characterization is still being refined. With no current ‘gold-standard’ method, commonly used pre-analytical protocols such as ultra-centrifugation harbor numerous limitations. For example, ultra-centrifugation often contaminates EV preparations with non-EV material such as soluble proteins; it can promote the EV aggregates and in turn alter the in vivo bio-distribution and function [105]. The isolation and characterization of EV methods have been extensively reviewed [96,106,107]. Despite encouraging results from preclinical studies showing diverse beneficial effects following MEx therapy across a range of pathologies [103], it is fair to conclude that the full potential of MEx therapy has been hindered by a lack of standardization in EV isolation and an incomplete understanding in MEx molecular composition and subsequent mechanism of action. Currently, the challenges of industrial scale-up, EV heterogeneity, and difficulties in defining metrics of MEx potency complicate the transition to clinical development [96]. Moreover, with such diverse biological actions, the pursuit for a ‘singular’ bioactive ingredient responsible for the beneficial effects of MEx is perhaps futile. Rather, the synergistic bombardment of multiple ‘bioactive’ components is a more likely culprit. However, until technological advances allow for a detailed characterization at a single exosome level, this remains a topic for debate.

## 7. Modulation of Macrophage Function: The Gatekeeper of Exosome-Based Therapeutics

**Figure 2:**
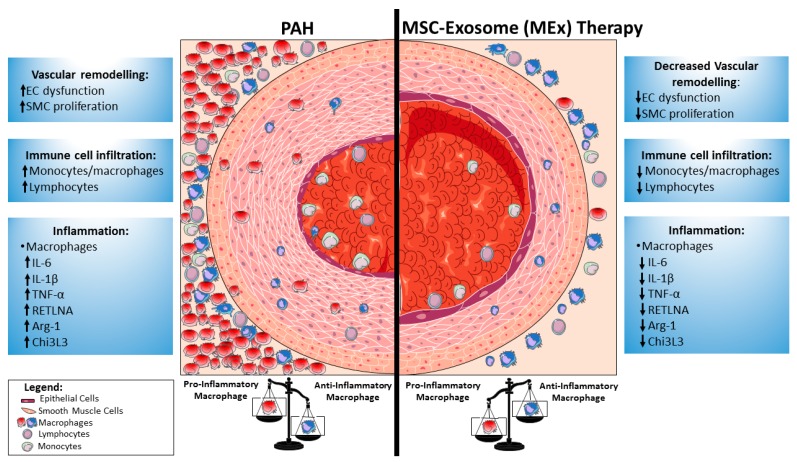
Modulation of macrophage function by mesenchymal stem cell (MSC)-exosomes (MEx): implications in pulmonary hypertension (PH). A diagrammatic illustration of the pulmonary vascular changes in pulmonary arterial hypertension (PAH) (**left**) and mechanisms by which MEx may afford their beneficial effects (**right**). In patients with PAH, pulmonary vessels are characterized by phenotypically altered, hyperproliferative smooth muscle cells (SMCs). Simultaneously, the blood vessels in PAH become decorated with an influx of inflammatory cells including, but not limited to, lymphocytes, monocytes, and proinflammatory macrophages. These invading inflammatory cells augment the inflammatory cascade by the secretion of pro-inflammatory and pro-fibrotic cytokines. MEx modulate macrophage phenotypes away from the pro-inflammatory phenotype towards that of a pro-resolving, anti-inflammatory state. Endothelial cell (EC); interleukin- (IL-); tumor necrosis factor alpha (TNF-α); arginase-1 (Arg-1); resistin-like alpha (Retnla); chitinase 3-like 3 (Chi3L3).

The exact mechanisms underlying the immunomodulatory functions of MEx remain elusive. It is well established that macrophages orchestrate both the initiation and resolution of inflammation. Thus, manipulation of monocyte trafficking and/or lung macrophage function represents an attractive target for emerging therapeutics, including stem cell-based approaches. Numerous studies have reported MEx as potent immunomodulators (for reviews see [110,111]). Herein, we speculate that macrophage immunomodulation is the ‘gatekeeper’ to the success of MEx therapies (Figure 2).

The early macrophage recruitment and activation seems to be necessary for the development of vascular disease, and, therefore, these processes serve as the best temporal window for exosome therapy [23,26]. We have previously noted that MEx blunt the pulmonary influx of macrophages in a hypoxia-PH model and that this was associated with a suppression in BALF inflammatory markers such as Il-6 and TNF-α [29]. More recently, in addition to ameliorating vascular remodeling, blood vessel loss and rescuing RVH, we found that a bolus IV dose of human MEx suppressed whole lung inflammation in experimental BPD [28]. Using an unbiased whole lung RNA-seq approach we found that MEx modulated the lung transcriptome, towards that akin to their normoxia (healthy control) counterparts. Gene ontology analysis suggested that hyperoxia promoted the induction of innate and adaptive-mediated inflammatory signals and that this was suppressed by MEx treatment. Building upon this, we also demonstrated that MEx modulate the macrophage phenotype fulcrum both in vitro and in vivo, as evidenced by the suppression of markers associated with the proinflammatory “M1-like” state (such as TNF-α, Il6, and Ccl2) and modulation of the expression levels of anti-inflammatory, pro-remodeling, “M2-like” states (including Cd206, Arginase-1, and Retnla). Similarly, using in vitro cell models and an in vivo model of cardiotoxin induced-skeletal muscle injury, Lo Sicco and colleagues demonstrated that adipose tissue derived-MSCs release EVs endowed with potent anti-inflammatory capacities to balance macrophage polarization toward an “M2-like” profile [112]. More recently, Hyvärinen and coworkers demonstrated that MEx enhance the anti-inflammatory phenotype of regulatory (‘M2-like’) macrophages by downregulating the production of interleukin (IL)-23 and IL-22 [113]. Of interest, other studies have found that exosomes isolated from MSCs preconditioned by lipopolysaccharide (LPS) may have superior ‘regulatory’ abilities for macrophage polarization and resolution of chronic inflammation by shuttling microRNA let-7b [114]. In turn, it is fair to speculate that the impact of MEx on pulmonary macrophage phenotypes underlies their therapeutic action through the modulation of pulmonary inflammation.

In a rat model of spinal cord injury, Lankford and coworkers assessed the tissue distribution and cellular targeting of fluorescently labeled MEx [115]. They reported that intravenously delivered MEx were detected in contused regions of the spinal cord, but not in the non-injured region of the spinal cord. Of interest, the “hotspots” where the labeled exosomes were detected were also associated with CD206-expressing ‘M2’ macrophages in the spinal cord. This was confirmed by co-localization with anti-CD63 antibody labeling, a tetraspanin characteristically expressed on exosomes. In accordance, our in vitro studies have already shown that MEx readily interact and are taken-up by macrophages [28]. Notwithstanding the limitations of dye-labeled exosomes and the technical difficulties in assessing the in vivo biodistribution of exogenous exosomes, studies such as Lankford et al., support the notion that MEx readily interact with macrophages in vivo and that the therapeutic effects are mediated by modulating macrophage function. Furthermore, several studies have also suggested that macrophages play an important role in the clearance of intravenously administered exosomes [116,117]. It is fair to speculate that as a core component of both innate and adaptive responses, macrophages readily interact with foreign particles/exosomes and that the ‘bioactive’ components of MEx, via mechanisms which are still to be elucidated, program the anti-inflammatory macrophage phenotype.

The precise bioactive component(s) for the beneficial effects of MEx remain unknown. Indeed, several reports have suggested that the integrins, proteins, fatty acids, and microRNAs are responsible for some of the therapeutic activity. Arguably, the largest body of evidence belongs to microRNAs, small, non-coding RNAs of ~22 to 26 nucleotides in length that function primarily as post-transcriptional regulators of a variety of pathophysiological processes. MicroRNAs have been shown to regulate macrophage polarization and subsequent effects on inflammation (reviewed in [118,119,120]). For example, miR-21, miR-155, miR-125b, and miR467b can push macrophages towards an “M1-like” phenotype, where miR-124, miR-142-5p, miR-146a, and miR-511 are associated with an “M2-like” state. Whether MEx harbor the precise microRNAs, and if so, at the relevant levels to achieve polarization, remains to be confirmed.

On balance, it is clear that MEx therapy ameliorates core features of experimental PH. However, the precise mechanism(s) responsible for such effects remain elusive. Are the beneficial effects of MEx merely due to modulation of macrophage phenotypes and monocyte trafficking? Other studies have shown that MSC-microvesicles (albeit a different EV subset that is larger than exosomes) can modulate the phagocytic capacity of macrophages, which subsequently affords protection in acute lung injury models, yet it remains unclear if the exosomal EV subtype can recreate this [93]. Indeed, further studies that actively address the issues raised in this manuscript are required as several questions remain unanswered. For example, what is the in vivo metabolic fate and bio-distribution of MEx? Do MEx exert beneficial actions in immunocompromised animals (i.e., macrophage depleted animals)? Do MEx equally affect the bone marrow, the lung-, and the circulating monocyte/macrophage lineages? How do MEx affect T lymphocyte function and number? Thus, carefully designed studies should explore (i) if/how MEx alter the epigenetic landscape of macrophages and (ii) the interaction of macrophages/monocytes preconditioned with MEx with other immune cell types.

## 8. Conclusions

An improved molecular understanding of the role macrophages play in immune responses relevant to PAH pathophysiology may pave the way for new therapeutic tools to effectively treat and reduce the risk of further complications. Here, we speculate that manipulation of lung macrophage function represents an attractive target for emerging therapeutic approaches. Exhibiting potent immunomodulatory capabilities, MSC-based approaches have been shown to be effective in ameliorating injury and/or reestablishing homeostasis in several preclinical models of lung disease relevant to PAH. Several studies have shown that the therapeutic capacity of MSCs is harnessed in their secretome, and more recent studies have demonstrated that the ‘major’ therapeutic vector therein is represented by the exosomes. In turn, exosome-based therapeutics represent a most promising next generation approach for treating a diverse number of diseases, particularly diseases the pathogenesis of which involve a primary (or major) inflammatory component. Although more studies are needed to address this issue, in this review, we postulate that MEx modulate macrophage function, which in turn, is responsible, at least in part, for the beneficial effects of MEx treatment in experimental models relevant to PAH.

## Figures and Tables

**Table 1:** Application of MEx therapy in models relevant to PAH.

Disease	Species	MSC-Product ‘Nomenclature’	Final Isolation Step	Dose Assessment	Dose	Ref
Hypoxia-PH	Mouse	Exosomes	PEG-SEC	Protein	0.1–10 μg	[29]
MCT-PH	Mouse	Exosomes	UC (100,000× *g*)	Protein	25 μg	[104]
MCT-PH	Rats	Microvesicles	UC (100,000× *g*)	Protein	30 μg	[108]
Hyperoxia-BPD	Mouse	Exosomes	Density Cushion	Cell equivalent	0.5 × 10^6^	[28]
Hyperoxia-BPD	Mouse	Extracellular vesicles	UC (110,000× *g*)	Cell equivalent	0.7 × 10^6^	[109]

UC: ultracentrifugation. PH: pulmonary hypertension. BPD: bronchopulmonary dysplasia. PEG-SEC: polyethylene glycol-size exclusion chromatography.
